# Associations of body composition with neoadjuvant chemotherapy response, toxicity, and prognosis in breast cancer: a scoping review

**DOI:** 10.1007/s00280-026-04940-7

**Published:** 2026-08-12

**Authors:** Samantha Ligertwood, Femi E. Ayeni, Andrew Parsonson, Senarath Edirimanne

**Affiliations:** 1 Sydney Medical School, The University of Sydney, Sydney, Australia; 2 Nepean Institute of Academic Surgery, The University of Sydney, 62 Derby St, Penrith, NSW 2750 Australia; 3 Department of Medical Oncology, Nepean Hospital, Penrith, NSW Australia

**Keywords:** Breast cancer, Body composition, Sarcopenia, Adiposity, Prognosis, Pathological response, Survival, Skeletal muscle, Adipose tissue, Visceral fat, Subcutaneous fat

## Abstract

**Background:**

Breast cancer is the leading cause of cancer-related death in women worldwide. There is increasing interest in using detailed body composition parameters, such as skeletal muscle mass and adiposity, to better predict treatment response, toxicity, and survival in breast cancer patients receiving neoadjuvant chemotherapy (NAC). This study synthesised current literature on the effect of body composition in breast cancer patients undergoing NAC and identified gaps for future research.

**Methods:**

A scoping review was conducted in accordance with PRISMA-ScR and Arksey & O’Malley frameworks. Embase, MEDLINE, PubMed, and Google Scholar were searched for studies published between 2010 and 2025 to map available evidence. Eligible studies included female breast cancer patients receiving NAC with body composition assessment and at least one outcome of response, toxicity, or prognosis.

**Results:**

Twenty studies met inclusion criteria. Computed tomography at the L3 vertebral level was the most common assessment method. Findings were heterogeneous; 3 studies reported a statistically significant association between sarcopenia and reduced pathological response, while results for adiposity were inconsistent. Half of the studies assessing toxicity reported higher toxicity rates in patients with sarcopenia and/or adiposity. Prognostic findings were variable, with some studies reporting associations with visceral adiposity, and only one study reporting a significant association with sarcopenia.

**Conclusions:**

Evidence regarding the relationship between adiposity and NAC toxicity and prognosis in breast cancer remains suggestive, while findings for sarcopenia are inconsistent. Prospective, standardised studies are needed to clarify the role of body composition in breast cancer outcomes and its relevance in treatment planning.

**Supplementary Information:**

The online version contains supplementary material available at 10.1007/s00280-026-04940-7.

## Introduction

Breast cancer is the most prevalent cancer, and the leading cause of cancer-related death, in women worldwide [1]. According to the World Health Organisation (WHO), there were 2.3 million new cases of female breast cancer in 2022, with 670,000 women losing their life to the disease [2]. While mortality rates have been down trending, breast cancer survivors are facing an increasing disease burden, necessitating further research into the risk factors for poor prognoses and negative treatment outcomes [3]. The influence of body composition on breast cancer treatment outcomes, and therefore its use as a prognostic tool, is one particular area of interest currently [4]. 

The assessment of body composition has developed greatly in recent years, largely due to the technological advancements in imaging modalities used [5]. In the past, body mass index (BMI) was the main tool commonly used to assess body size and its association with certain diseases [6]. In the context of breast cancer, the relationship between BMI and mortality has been widely researched, albeit with mixed results [7–10]. A 2011 prospective study collected data on the BMI of breast cancer patients prior to diagnosis, and investigated the relationship between BMI and all-cause mortality [9]. The greatest risk of overall mortality was found in morbidly obese (BMI $\:\ge\:$40 kg/m^2^) and underweight (BMI <18.5 kg/m^2^) patients, while other weight ranges were not associated with significantly increased risks [9]. This was contradicted, however, by a meta-analysis of 82 studies, which found that both obesity (BMI > 30 kg/m^2^) and being overweight (25–29.9 kg/m^2^) were associated with increased mortality risk, alongside being underweight [7]. Conversely, a pooled analysis investigating BMI and survival across several cancer types did not find any relationship between BMI and survival in breast cancer [8]. While this analysis may have been underpowered [8], the association of mortality and BMI classifications remains mixed in previous studies [7, 9]. 

Caan et al. [10] theorised these discrepancies may result from BMI masking levels of muscle mass, which may be low in a higher BMI, and levels of adiposity, which may be high despite a low BMI. This was supported in a 2018 observational study, which demonstrated that survival is more strongly associated with muscle mass and adiposity than BMI, with BMI having no significant relationship to overall mortality. [10] In comparison, a low muscle mass, or sarcopenia, and high total adipose tissue were both associated with increased mortality risk. [10] In particular, sarcopenic obesity, the mixed condition of obesity masking sarcopenia, is an independent factor in solid tumour and breast cancer prognosis, causing a hazard ratio for survival four times higher in patients with sarcopenic obesity compared to those without [6]. 

Currently, chemotherapy drugs are dosed according to patient weight and predicted body surface area (BSA) [11]. However, in early breast cancer patients who received either neoadjuvant or adjuvant chemotherapy, it was observed that sarcopenia was associated with a greater risk of chemotherapy toxicity than that of non-sarcopenic patients, even after controlling for age, comorbidities and BMI [12]. These findings support previous research by Shachar et al. [13], which investigated the relationship between body composition and toxicity related to anthracycline and taxane–based chemotherapy in early breast cancer patients.

While there is support for the role of sarcopenia in chemotherapy toxicity, the impact of adiposity has varied in the literature [13–15]. The previously discussed research by Shachar et al. found no association between measures of adipose tissue, such as subcutaneous adipose tissue (SAT) and visceral adipose tissue (VAT), and any grade 3/4 toxicity [13]. However, an earlier 2014 study found that in advanced breast cancer patients treated with doxorubicin, an increased risk of haematological toxicities occurred with higher intra-abdominal fat volumes, though these findings are limited by the small sample size of 84 patients investigated [15]. Comparison between these studies is difficult though, due to variations in the measure of adiposity and toxicity outcomes used, and further research on the topic may be beneficial.

It is on this premise the current study scoped the literatures widely to examine the effect of body composition on neoadjuvant chemotherapy response, toxicity, and prognosis in breast cancer patients. The aims of this study are to synthesise current literature; to establish the components of body composition and its measurement techniques in breast cancer patients; identify the associations between body composition and the outcomes of neoadjuvant chemotherapy in breast cancer, such as pathological response to treatment, chemotherapy toxicity, and prognosis; and determine if any gaps are present in the existing literature which may present potential areas for future study.

## Methods

### Study design

The study employed The Preferred Reporting Items for Systematic reviews and Meta-Analyses (PRISMA) extension for scoping reviews [16], along with the Arksey and O’Malley methodological framework for scoping studies [17].

### Eligibility criteria

Papers were included in this review if they: were published between 2010 and 2025; involved female breast cancer patients undergoing NAC; and included results that were relevant to one or more of the outcomes of pathological response, chemotherapy toxicity, and/or prognosis. Studies were excluded if they: investigated patients undergoing adjuvant chemotherapy and did not include a separate analysis of NAC patients; investigated BMI instead of body composition; investigated male breast cancer patients; did not investigate at least one of the outcomes of pathological response, toxicity, or prognosis; or did not contain sufficient results on these outcomes for data extraction. Papers written in the form of study protocols, review articles, editorials and commentaries were also excluded from this review.

### Sources

To gather and select the relevant literature, the bibliographic databases of Embase, Google Scholar, MEDLINE and PubMed were searched. All searches were limited to include only papers from January 2010 to May 2025. The search strategies were drafted and refined by the authors of this review (Appendix 1). The search results from each database were exported to EndNote, and then imported into Covidence, an online tool that can be used in the systematic and scoping review process. No additional database-specific search filters (e.g., language or study design) were applied beyond the predefined publication date range, to maximise the sensitivity of the search.

### Data extraction

A template for data extraction was developed by the author of this review to identify the relevant variables for extraction. This template was continuously updated throughout the extraction process to ensure all pieces of relevant information were included. The data was extracted independently by the author, SL and verified by FEA. Data was extracted based on the characteristics of each article; the outcomes investigated (e.g., response, toxicity, prognosis, including measures used and relevant definitions/cut-off values), assessment techniques (e.g., imaging modality, body composition assessed and relevant definitions/cut-off values), participant characteristics (e.g., cancer stage, breast cancer subtype, NAC regiment), and results relevant to each outcome. Chemotherapy toxicity was extracted as reported in individual studies. Toxicity assessment varied across studies and included measures such as treatment-related adverse events, hematologic toxicity, dose reductions, and treatment delays; however, specific parameters and grading systems were not consistently defined or reported. Adiposity was defined variably across studies based on study-specific metrics, including visceral adipose tissue, subcutaneous adipose tissue, and total fat area, typically derived from imaging modalities such as CT or PET-CT. The full data extraction template can be found in Appendix 2.

### Data handling

Studies were grouped by the outcome they investigated (e.g., response, toxicity, prognosis), and key study characteristics, participant characteristics, relevant definitions and broad findings were tabulated and summarised. All outcomes were extracted irrespective of statistical significance; however, the narrative synthesis emphasised statistically significant associations to highlight potential signals of effect.

## Results

### Study selection

**Fig. 1:**
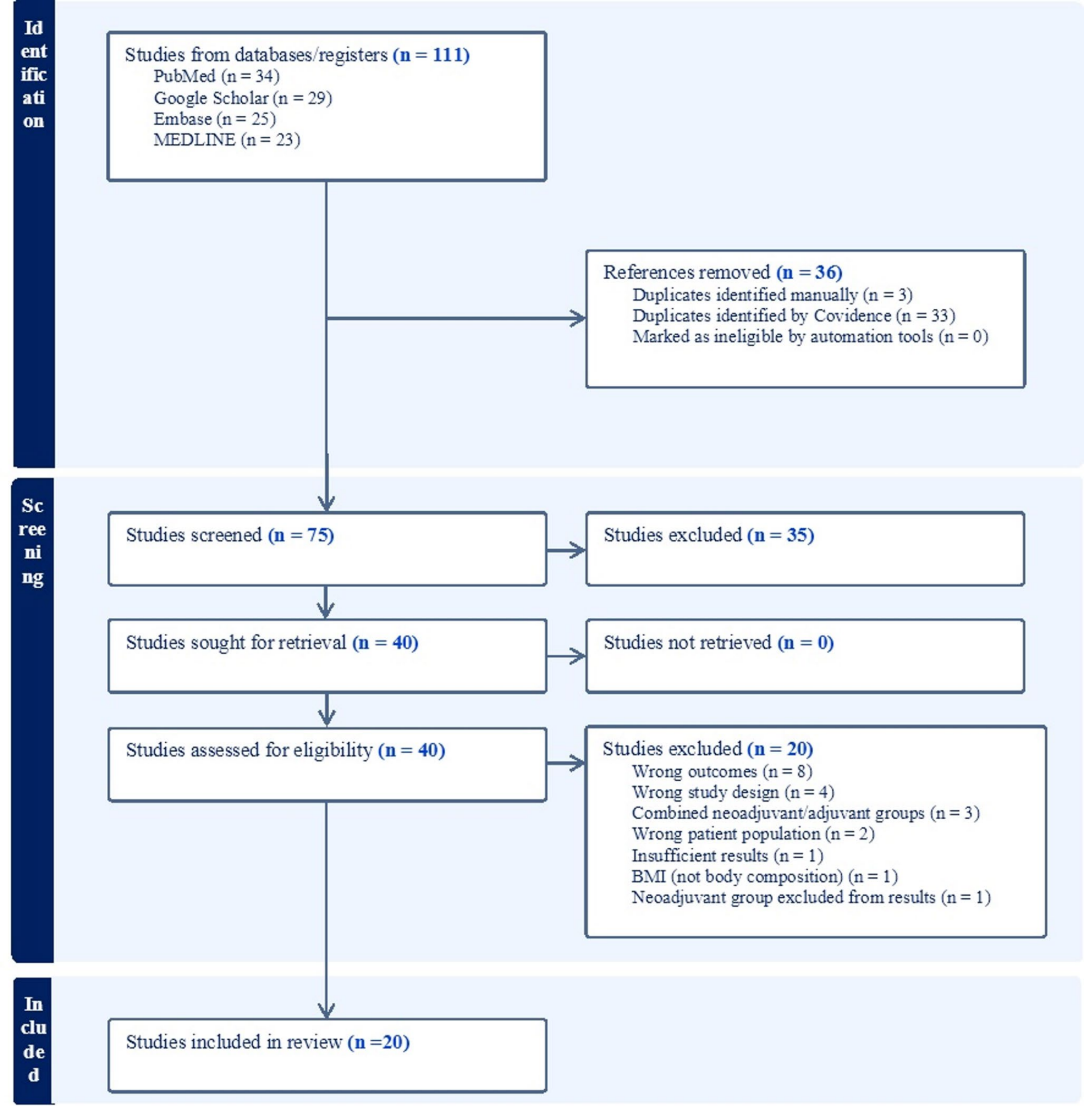
PRISMA flowchart of review process

The study screening and selection process is shown in Fig. 1. The search strategies identified a total of 111 studies, including 34 studies on PubMed, 29 studies on Google Scholar, 25 studies on Embase, and 23 studies on MEDLINE. After removal of 36 duplicate records, 75 studies underwent screening. Full text review was conducted on 40 studies, of which 20 met the inclusion criteria and were included in the final analysis [18–37]. 

### Study characteristics

Of the 20 studies examining the relationship between body composition and NAC outcomes in breast cancer, six investigated pathological response only [22–24, 29, 32, 36], four examined NAC toxicity alone [20, 27, 35, 37], and eight assessed both pathological response and prognosis [18, 19, 21, 25, 26, 31, 33, 34], while two studies evaluated all three outcomes [28, 30]. Overall, sixteen studies assessed pathological response, six assessed toxicity, and ten assessed prognosis following NAC. The key characteristics of included studies, including the body composition assessment, methods approach, and timing, are summarised in Table 1.

**Table 1:** Main characteristics and findings of included studies

Study ID	Title	Country	Study design	Participants (N=)	Prognosis measure	Toxicity measure	Assessment timing	Assessment method	Component assessed	Follow-up period	Findings
Outcome(s): pCR only											
Dulgar 2023 [22]	Is adipose tissue metabolic activity a predictor of pathological responses to neoadjuvant treatment in breast cancer.	Turkey	Retrospective cohort study	116			Pre- and post-NAC	PET-CT *(level not specified)*	SAT VAT V/S ratio	*Not specified.*	Statistically significant relationship between good PR and a higher VAT SUV (*P* = 0.037) and higher density of VAT (*P* = 0.043). SAT volume and metabolic activity had no significant impact on pCR. High V/S ratio was significantly associated with a good PR (*P* = 0.003). In multivariate analysis, V/S ratio (*P* = 0.02) was independently associated with pCR.
Guo 2024 [23]	Pretreatment Sarcopenia and MRI-Based Radiomics to Predict the Response of Neoadjuvant Chemotherapy in Triple-Negative Breast Cancer	China	Retrospective cohort study	121			Pre-NAC	CT L3 MRI	SMA SMI SFA SFT MMA	*Not specified.*	Only sarcopenia had a significant correlation with pCR grade ([OR], 0.4151 (95% CI: 0.1858-0.9274); *p* < 0.05). Sarcopenia also showed a correlation with pCR/non-pCR in the dataset ([OR], 0.3360 (95% CI: 0.1407-0.8022); *p* < 0.05).
Isıklar 2024 [24]	The Relationship Between Body Composition and Pathological Response to Neoadjuvant Chemotherapy in Breast Cancer Patients	Turkey	Retrospective, cross-sectional, observational study	69			At diagnosis	CT L3 PET-CT L3	SMA SMI	28 months (median)	The effect of SMI on pCR failure was not significant. Sarcopenia appeared to negatively affect pCR but was not statistically significant.
Lee 2021 [29]	Association between skeletal muscle loss and the response to neoadjuvant chemotherapy for breast cancer	Korea	Retrospective cohort study	246			Pre- and post-NAC	CT L3 CT T4	SMA SATI	28.85 months (median)	Skeletal muscle loss at both pre-NAC and post-NAC was associated with poor response. SATI, both pre- and post-NAC, had poor specificity and sensitivity in predicting pCR.
Rossi 2023 [32]	Muscle mass loss in breast cancer patients of reproductive age (≤45 years) undergoing neoadjuvant chemotherapy	Italy	Retrospective cohort study	52			Pre- and post-NAC	MRI	PecMA	158 days (± 25.5)	No significant differences in PMA values between complete and partial responses found.
Yirgin 2020 [36]	Comparison between body composition parameters and response to neoadjuvant chemotherapy by using pre-treatment PET CT in locally advanced breast cancer	Turkey	Retrospective cohort study	186			Pre-NAC	PET-CT L3	SAT VAT MT	*Not specified.*	No statistically significant correlation between pCR and SAT, VAT, MT (p:0,19, p:0,45, p:0,83). Very low correlation between SAT and more than 30% response (non-remarkable). No correlation between body composition parameters and pCR and non-pCR groups.
Outcome(s): Toxicity only											
Chen 2024 [20]	Prediction of hematologic toxicity in luminal type breast cancer patients receiving neoadjuvant chemotherapy using CT L1 level skeletal muscle index.	China	Retrospective cohort study	140		Haematologic toxicities occurring during NAC.	Pre-NAC	CT L3 CT L1	SMA SMI IMFA IMFI SFA SFI VFA VFI	*Not specified.*	Univariate analysis found statistically significant associations between SMA, SMI, SFA, SFI, VFA, and VFI with hematologic toxicity during NAC. Multivariate analysis demonstrated that an SMI<32.91 cm2/m2 was an independent risk factor for severe hematologic toxicity during NAC.
Di Leone 2025 [35]	The role of body composition in neurological and hematologic toxicity in a retrospective analysis of 120 breast cancer patients undergoing neoadjuvant chemotherapy: the COMBOTOX study.	Italy	Retrospective cohort study	120		(1) Main chemo-related toxicities (haematologic and neurological) (2) Common side effects related to chemotherapy, assessed in clinical interviews by oncologists before every cycle of chemotherapy, and recorded in patient reports. (3) Treatment interruptions due to toxicity	*Not specified.*	BIA	FM FFM *[both in kilograms (kg) and percentage (%)]*	*Not specified.*	A lower FM was as a protective factor against neurological toxicity, but this was not statistically significant. (OR 0.95, 95%CI 0.91-1.00; *p*=0.058). No other body composition parameter significantly affected the onset of hematologic toxicity. In those receiving an anthracycline-taxane plus carboplatin NAC regimen (*n*=25), there was a suggestive association that low FFM % negatively influenced hematologic toxicity (IOR 1.21, 95%CI 0.99-1.46, *p*=0.056), while a low FM% was significantly protective (IOR 0.82, 95%CI 0.67-0.99; *p*=0.041). No significant association was observed between body composition and chemotherapy interruptions related to toxicity or non-response. Reduced FM had a suggestive but not significant association as a protective role against hot flashes during NAC (OR 0.90, 95%CI 0.79-1.02; *p*=0.091), while a lower FFM% seemed to promote their onset (OR 1.11, 95%CI 0.98-1.26; *p*=0.093). No other associations were observed between body composition and adverse NAC events.
Jang 2023 [27]	Hematologic toxicities, sarcopenia, and body composition change in breast cancer patients undergoing neoadjuvant chemotherapy.	Korea	Retrospective cohort study	298		Haematologic toxicities occurring during the entire period of NAC.	Pre- and post-NAC	CT L3	SMA SMI SFI	5 months (mean)	Significantly different prevalence of anaemia between the sarcopenia and non-sarcopenia groups (*p* < 0.001) after NAC, with the sarcopenia group having an anaemia prevalence (*n* = 14) double that of the non-sarcopenia group (*n* = 7). No significant difference in the prevalence of neutropenia or thrombocytopenia between the two groups after NAC. Those with anaemia had a significantly decreased SMI and SFI after NAC (t(296) = 3.28, *p* = 0.0013; and t(296) = 3.405, *p*= 0.0008, respectively). No significant difference in visceral fat index (VFI) was found between the groups with or without anaemia. No significant difference in body composition changes were found between the groups with or without thrombocytopenia. Only VFI showed a significant difference between the two groups with or without neutropenia (t(296) = -2.082, *p* = 0.038).
van den Berg 2019 [37]	Body composition is associated with risk of toxicity-induced modifications of treatment in women with stage I-IIIB breast cancer receiving chemotherapy.	The Netherlands	Observational study	172		Toxicity-induced modifications to treatment.	Pre-NAC *(or during first chemotherapy cycle).*	DEXA	FM LM *[both as absolute in kilograms (kg) and relative as percentage (%) of total body weight]*	*Not specified.*	Those with a toxicity-induced modification of treatment had a higher FM and lower %LM compared to those without modifications. Higher absolute and relative FM was associated with a higher risk of modifications [HR per 5 kg fat mass: 1.14 (95%CI 1.04-1.25); HR per 5% increase in fat mass: 1.21 (95% CI 1.05-1.38)]. Higher relative LM was associated with a lower risk of toxicity-induced treatment modifications [HR per 5% increase in lean mass: 0.83 (95% CI 0.72-0.96)]. Absolute LM was not associated with treatment modifications. Low LM in combination with high FM was associated with a higher risk of toxicity-induced treatment modifications compared to having a normal LM in combination with normal FM (HR 1.33, 95% CI 1.01-1.75).
Outcome(s): pCR and prognosis											
Amitani 2022 [18]	Skeletal muscle loss during neoadjuvant chemotherapy predicts poor prognosis in patients with breast cancer.	Japan	Retrospective cohort study	141	OS DFS		Pre-NAC	CT L3 PET-CT L3	SMA SMI	70 months (median; range 3-131).	Increased SMI (>3% increase) group had a significantly higher pCR rate (*p* = 0.03) than the maintained (-3 to 3% change) and decreased SMI (>3% decrease) groups.
											A significantly poorer DFS was observed in the decreased SMI group compared to the maintained and increased groups (hazard ratio [HR] 8.29, 95% confidence interval [CI] 3.63-18.9, *p* < 0.001 for the decreased vs. increased SMI group; HR 3.49, 95% CI 1.65-7.36, *p* < 0.001 for the decreased vs. maintained SMI group), regardless of NAC regimen. A significantly reduced OS was also observed in the decreased SMI group (HR 19.8, 95% CI 6.43-60.6, *p* < 0.001 for the decreased vs. increased SMI group; HR 3.41, 95% CI 1.29-8.95, *p*=0.013 for the decreased vs. maintained SMI group). A decrease in SMI was significantly associated with poorer DFS on univariate analysis (HR 3.46, 95% CI 1.56-7.65, *p*<0.01), and was an independent predictive factor for poorer DFS on multivariate analysis (HR 3.68, 95% CI 1.54-9.01, *p*<0.01).
Tahtaci 2023 [33] ***N.B. Abstract only***	Impact of PET-CT-determined sarcopenia on survival and pathological complete response in breast cancer patients with neoadjuvant chemotherapy.	Turkey	Retrospective cohort study	23	OS		Pre- and post-NAC	PET-CT L3	SMI VFA Sarcopenic obesity	*Not specified.*	No association was found between sarcopenia and pCR.
											No association between sarcopenia and OS. Survival was significantly associated with both high VFA and sarcopenic obesity post-NAC. A high VFA post-NAC was significantly associated with increased mortality in multivariate analysis (HR: 3.278 CI: 95% (1.143-9.401), *p*=0.027).
Omarini 2019 [31]	Predictive Role Of Body Composition Parameters In Operable Breast Cancer Patients Treated With Neoadjuvant Chemotherapy	Italy	Retrospective cohort study	137	OS RFS		Pre-NAC	CT L3	LMCA SFA VFA L/S ratio	*Not specified.*	High VFA and L/S ratio (indicative of liver steatosis) were negative predictive factors for pCR (*p*≤0.05).
											No significant association was observed between body composition parameters and either OS or RFS.
Iwase 2021 [25]	The Prognostic Impact of Body Composition for Locally Advanced Breast Cancer Patients Who Received Neoadjuvant Chemotherapy	United States	Retrospective cohort study	198	OS DFS RFS		Pre-NAC	CT L3	SMI SAT VAT VAT-HU V/S ratio	4.7 years (median)	VAT, VAT-HU, and SAT had no significant effect on pCR (*p* = 0.62; *p* = 0.56; *p* = 0.19).
											A low V/S ratio (<34) was significantly associated with shorter OS using a proportional hazards assumption. A V/S ratio <34 was associated with DFS, RFS and OS (*p* = 0.001; *p* < 0.001; *p* = 0.026 per log-rank test). Patients with a V/S ratio < 34, compared to those with a V/S ratio ≥ 34, had a shorter OS and a higher risk of an event, including recurrence or death, during follow-up. No other body composition measurements demonstrated significant prognostic value.
Carbognin 2020 [19] ***N.B. Abstract only***	Prognostic impact of body composition (BC) changes during neoadjuvant chemotherapy (NACT) in breast cancer patients	Italy	Retrospective cohort study	93	DFS		At diagnosis and post-NAC	CT L3	SMM IMAT SAT VAT	40 months (median; range 14-76)	Apart from an observed trend with baseline SMM (*p*=0.078), body composition parameters were not associated with pCR.
											A change in VAT during NAC was an independent prognostic factor for DFS (HR 7.48, 95% CI 0.91-60.5, *p*=0.061). Patients who gained VAT >12 cm2 during NAC had a significantly worse DFS than those who either had a decrease in VAT or gained VAT ≤12 cm2 (3-year DFS 79.8% vs. 95.7%, *p*=0.03, respectively).
Iwase 2016 [26]	Impact of body fat distribution on neoadjuvant chemotherapy outcomes in advanced breast cancer patients	Japan	Retrospective cohort study	172	DDFS		Pre-NAC *(at time of initial treatment)*	CT L3	SMA SMI SFA VFA L/S ratio	1638 days (median)	Body composition parameters were not associated with pCR.
											The high VFA group (VFA ≥ 100 cm2) had a significantly worse DDFS than the low VFA group (VFA < 100 cm2) (log-rank test; *P* < 0.05, HR: 2.36, 95% CI: 1.27-4.38). VFA was an independent prognostic factor for DDFS on multivariate analysis (*P* < 0.05, HR: 2.42, 95% CI: 1.28-4.57). No significant difference in DDFS was observed in relation to SFA, LSMI, or L/S ratio. No significant differences in OS were observed across body composition parameters.
Del Fabbro 2012 [21]	The Relationship Between Body Composition and Response to Neoadjuvant Chemotherapy in Women with Operable Breast Cancer	United States	Retrospective cohort study	129	OS PFS		Pre-NAC	CT L3	SMI SAT VAT	7.74 years (median; range 0.17-10.38).	Sarcopenic status (*n*=18) was not significantly associated with pCR (*p*= 0.0711). However, a trend towards a higher rate of response was observed in those with sarcopenia (OR, 2.74; 95% CI, 0.92- 8.22). Sarcopenia was of borderline significance in predicting pCR (*p* = 0.0508).
											Sarcopenia was not significantly associated with OS or PFS. However, SMI and VAT were associated with OS. For every unit increase in SMI, a 2% higher hazard for death was observed (hazard ratio [HR], 1.02; 95% CI, 1.00 -1.04; *p* = 0.0309). For every unit increase in VAT, a 0.6% higher hazard for death was observed (HR, 1.006; 95% CI, 1.001-1.012; *p* = 0.0193).
Trestini 2023 [34]	Predictive and prognostic effect of computed tomography derived body composition analysis during neoadjuvant chemotherapy for operable and locally advanced breast cancer.	Italy	Retrospective cohort study	93	DFS		At diagnosis and post-NAC	PET-CT L3	SMI SMM IMAT SAT VAT	47 months (median)	Body composition measurements had no significant effect on pCR. Higher SMM and lower IMAT at baseline were correlated with pCR (*P* = 0.084 and *P* = 0.070, respectively), however this was not significant.
											VAT change during NAC was significantly associated with DFS (HR, 10.2; 95% CI, 1.33 75.5; *P* = 0.026), and was an independent prognostic factor for DFS on multivariate analysis. Pts who gained ≥10% of VAT during NAC had significantly worse DFS than those who gained <10% of VAT (5yr DFS 71.4 vs. 96.3, *P* = 0.009, respectively).
Outcome(s): pCR, toxicity and prognosis											
Karaca 2024 [28]	Sarcopenia’s Role in Neoadjuvant Chemotherapy Outcomes for Locally Advanced Breast Cancer: A Retrospective Analysis.	Turkey	Retrospective cohort study	226	OS DFS	Presence of either thrombocytopenia or neutropenia during NAC.	Pre- and post-NAC	CT L3	PsoMA	37.2 months (median)	Neither pre- nor post-NAC sarcopenia influenced pCR. When comparing pre- and post-NAC sarcopenia, no significant differences in pCR were observed.
											No association was found between either pre- or post-NAC sarcopenia and recurrence or mortality. No significant differences in recurrence or mortality were observed when comparing pre- and post-NAC sarcopenia. No statistical differences in DFS or OS between pre- and post-NAC sarcopenic and non-sarcopenic patients were observed.
											No significant differences in haematologic adverse effects were observed between sarcopenic and non-sarcopenic patients.
Mun 2025 [30]	Skeletal Muscle Density as a Predictive Marker for Pathologic Complete Response in Triple-Negative Breast Cancer Treated with Neoadjuvant Chemoimmunotherapy	Korea	Retrospective cohort study	144	OS EFS PFS	Treatment discontinuation of at least one agent during the neoadjuvant phase.	At diagnosis	CT L3	SMA SMI SMD	5.2 months (median; 95% CI, 13.6 - 18.9 months).	While not statistically significant, a higher proportion of patients achieved pCR in the high-SMD group (63.2% vs. 44.1%, *p* = 0.066). *NACI group* (*n* = 102): No significant association between SMI and pCR. SMD was significantly associated with, and was an independent predictor of, pCR. The pCR OR was 1.11 for every 1-unit increase, 1.67 for every 5-unit increase, and 2.78 for every 10-unit increase in SMD (95% CI: 1.04-1.19, *p* = 0.003; 95% CI: 1.20-2.40, *p* = 0.003; 95% CI: 1.45-5.74, *p* = 0.003). A significant increasing trend was observed between SMD and pCR rates, with the high-SMD group demonstrating the highest rate of pCR, followed by the medium- and low-SMD groups (χ2 for trend, *p* = 0.028). *NAC group* (*n* = 42): No significant association was observed between pCR and SMD.
											*NACI group*: No significant association between SMI and EFS. EFS was significantly longer in the high-SMD group compared to the low-SMD group (*p* = 0.017, median EFS-not reached). PFS and OS were significantly longer in the high-SMD group compared to the low-SMD group. *NAC group*: EFS was not significantly different between SMD high and low groups (*p* = 0.850).
											*NACI group*: 15 patients (14.7%) discontinued due to adverse events. 4 of these patients completed NAC by ceasing pembrolizumab; all were in the high-SMD group. 11 patients ceased all NAC; 3 were in the high-SMD group, while 8 were in the low-SMD group. Thus, the low-SMD group had a significantly higher rate of total treatment discontinuation (4.4% vs. 23.5%, χ2 *p* = 0.003). There was a significant positive correlation between SMD and RDI (Spearman’s ρ = 0.31, *p* = 0.002). No significant correlation was seen between SMI and RDI.

BIA, bioelectrical impedance analysis; CI, confidence interval; CT, computed tomography; DEXA, dual x-ray absorptiometry; DFS, disease free survival; DDFS, distant disease-free survival; EFS, event-free survival; FM, fat mass; FFM, fat-free mass; IMAT, intramuscular adipose tissue; IMFI, intermuscular fat index; LM, lean mass; LMCA, lumbar muscle cross-sectional area; L/S ratio, liver/spleen ratio (surrogate for liver steatosis/internal fat distribution); MRI, magnetic resonance imaging; NAC, neoadjuvant chemotherapy; NACI, neoadjuvant chemoimmunotherapy; OR, odds ratio; OS, overall survival; pCR, pathological complete response; PET-CT, positron emission tomography–computed tomography; PR, pathological response; PFS, progression-free survival; PecMA, pectoralis muscle area; PsoMA, psoas muscle area; RDI, relative dose intensity; RFS, recurrence-free survival; SAT, subcutaneous adipose tissue; SATI, subcutaneous adipose tissue index; SFA, subcutaneous fat area; SFI, subcutaneous fat index; SFT, subcutaneous fat thickness; SMA, skeletal muscle area; SMD, skeletal muscle density; SMI, skeletal muscle index; SMM, skeletal muscle mass; VAT, visceral adipose tissue; VAT-HU, visceral adipose tissue Hounsfield unit (indicative of VAT quality); VAT SUV, visceral adipose tissue standardized uptake value; VFA, visceral fat area; VFI, visceral fat index; V/S ratio, VAT to SAT ratio.

Italy and Turkey contributed the highest number of studies (*n* = 5 each), with additional studies conducted in Korea, China, Japan, United States and The Netherlands. Most studies were retrospective in design, with only one prospective observational [37]. Only four studies were published prior to 2020 reflecting interest in this field [26, 31, 21, 37] Sample sizes varied, with three studies including more than 200 participants [27–29], five including fewer than 100 participants [19, 24, 32–34], and the remaining studies including between 100 and 200 participants. [18, 20–23, 25, 26, 30, 31, 35, 36, 37].

In the assessment of body composition (Table 2), most studies used CT or PET-CT, [1–10, 12, 13, 15, 18–22, 24–31, 33, 34, 36–43] while two used MRI [23, 32], one used DEXA [35], and one used bioelectrical impedance analysis (BIA) [11]. Twelve studies assessed body composition at a single time point prior to NAC [18, 20, 21, 23–26, 30, 31, 37], whereas eight studies performed assessments both before and after completion of NAC [19, 22, 27–29, 32–34]. 

**Table 2:** Methods used to assess body composition in selected studies

Assessment method		Study
CT	L1	Chen 2024 [20]
	L3	Amitani 2022 [18] Carbognin 2020 [19] Chen 2024 [20] Del Fabbro 2012 [21] Guo 2024 [23] Iwase 2016 [26] Iwase 2021 [25] Isıklar 2024 [24] Jang 2023 [27] Karaca 2024 [28] Lee 2021 [29] Mun 2025 [30] Omarini 2019 [31]
	T4	Lee 2021 [29]
PET-CT	L3	Amitani 2022 [18] Isıklar 2024 [24] Tahtaci 2023 [33] Trestini 2023 [34] Yirgin 2020 [36]
	*Unspecified level*	Dulgar 2023 [22]
MRI		Guo 2024 [23] Rossi 2023 [32]
BIA		Di Leone 2025 [35]
DEXA		van den Berg 2019 [37]

BIA, bioelectrical impedance analysis; CT, computed tomography; DEXA, dual x-ray absorptiometry; L1, first lumbar vertebrae; L3, third lumbar vertebrae; L4, fourth lumbar vertebrae; MRI, magnetic resonance imaging; PET-CT, positron emission tomography–computed tomography; T4, fourth thoracic vertebrae

Skeletal muscle quantity and sarcopenia were mostly assessed using skeletal muscle index (SMI) at the L3 vertebral level. CT-based assessment at L3 was the most frequently reported approach [18–21, 23–31], with several studies also utilising PET CT at the same anatomical level [18, 24, 33, 34, 36]. Overall, the L3 vertebral level was the most commonly used site for body composition assessment; however, variation existed in both anatomical level and measurement approach. One study assessed skeletal muscle at the L1 [20], and another at the T4 level [29]. Alternative measures were also reported, including psoas muscle area [28], lean mass [37], and muscle tissue area [36], while only one study assessed skeletal muscle quality using skeletal muscle density (SMD) [30]. 

Similarly, adiposity was variably defined across studies using a range of metrics, contributing to overall heterogeneity. Most studies assessed adiposity using measures of subcutaneous and visceral fat, including subcutaneous adipose tissue (SAT), visceral adipose tissue (VAT), subcutaneous fat area (SFA), and visceral fat area (VFA). Only two studies used fat mass (FM), measured by BIA or DEXA, as a measure of adiposity.[ 35,37] Internal fat distribution was assessed in two studies using the presence of hepatic steatosis quantified by the liver-to-spleen (L/S) ratio [26, 31]. 

## Main outcome findings

The narrative synthesis summarised reported associations between body composition and neoadjuvant chemotherapy (NAC) outcomes, including pathological response (Table 3), toxicity (Table 4), and prognosis (Table 5). Although some studies reported statistically significant associations, findings were heterogeneous and frequently not replicated across studies.

**Table 3:** Significant effects of sarcopenia and adiposity on pathological response

Study	Sarcopenia		Adiposity	
	Relevant cut-off value	Effect	Relevant cut-off value	Effect
Amitani 2022 [18]	SMI decrease > 3%	Reduced pCR		
Carbognin 2020 [19]	*Continuous variable(s)*	No effect	*Continuous variable(s)*	No effect
Del Fabbro 2012 [21]	SMI ≤ 38.5	No effect		
Dulgar 2023 [22]			*Continuous variable(s)*	Improved pCR
Guo 2024 [23]	SMI < 39	Reduced pCR	*Continuous variable(s)*	No effect
Isıklar 2024 [24]	SMI ≤ 38.5	No effect		
Iwase 2016 [26]	*Not specified.*	No effect	SFA ≥ 100 VFA ≥ 100 L/S ratio > 1	No effect
Iwase 2021 [25]			V/S ratio ≥ 34	No effect
Karaca 2024 [28]	PsoMA 415.4	No effect		
Lee 2021 [29]	SMI (T4) 6	Reduced pCR	*Continuous variable(s)*	No effect
Mun 2025 [30]	*Continuous variable(s)*	No effect		
Omarini 2019 [31]	SMI < 38.5	No effect	SFA ≥ 100 VFA ≥ 100 L/S ratio < 1	Reduced pCR
Rossi 2023 [32]	*Continuous variable(s)*	No effect		
Tahtaci 2023 [33]	SMI 38.9	No effect		
Trestini 2023 [34]	SMI < 41	No effect	*Continuous variable(s)*	No effect
Yirgin 2020 [36]	*Continuous variable(s)*	No effect	*Continuous variable(s)*	No effect

L/S ratio, liver/spleen ratio (cm^2^/m^2^); pCR, pathological complete response; PsoMA, psoas muscle area (mm^2^); SFA, subcutaneous fat area (cm^2^); SMI, skeletal muscle index (cm^2^/m^2^); VFA, visceral fat area (cm^2^); V/S ratio, visceral adipose tissue to subcutaneous adipose tissue ratio

**Table 4:** Significant effects of sarcopenia and adiposity on NAC toxicity

Study	Sarcopenia		Adiposity	
	Relevant cut-off value	Effect	Relevant cut-off value	Effect
Chen 2024 [20]	SMI (L3) < 39 SMI (L1) < 32.91	Increased toxicity	IMFA ≥ 3.59 IMFI ≥ 1.35 SFA ≥ 155.25 SFI ≥ 56.90 VFA ≥ 112.55 VFI ≥ 34.78	Increased toxicity
Di Leone 2025 [35]	*Continuous variable(s)*	No effect	*Continuous variable(s)*	No effect
Jang 2023 [27]	SMI < 38.5	Increased anaemia	*Continuous variable(s)*	Increased neutropenia
Karaca 2024 [28]	PsoMA 415.4	No effect		
Mun 2025 [30]	*Continuous variable(s)*	No effect		
van den Berg 2019 [37]	*Continuous variable(s)*	Increased toxicity	*Continuous variable(s)*	Increased toxicity

IMFA, intermuscular fat area (cm^2^); IMFI, intermuscular fat index (cm^2^/m^2^); PsoMA, psoas muscle area (mm^2^); SFA, subcutaneous fat area (cm^2^); SFI, subcutaneous fat index (cm^2^/m^2^); SMI, skeletal muscle index (cm^2^/m^2^); VFA, visceral fat area (cm^2^); VFI, visceral fat index (cm^2^/m^2^)

**Table 5:** Significant effects of sarcopenia and adiposity on prognosis

Study	Sarcopenia		Adiposity	
	Relevant cut-off value	Effect	Relevant cut-off value	Effect
Amitani 2022 [18]	SMI decrease > 3%	Worsened prognosis		
Carbognin 2020 [19]	*Continuous variable(s)*	No effect	VAT > 12	Worsened prognosis
Del Fabbro 2012 [21]	SMI ≤ 38.5	No effect	*Continuous variable(s)*	Worsened prognosis
Iwase 2016 [26]	*Not specified.*	No effect	L/S ratio > 1	Worsened prognosis
Iwase 2021 [25]	*Continuous variable(s)*	No effect	V/S ratio ≥ 34	Worsened prognosis
Karaca 2024 [28]	PsoMA 415.4	No effect		
Mun 2025 [30]	*Continuous variable(s)*	No effect		
Omarini 2019 [31]	LSMI < 38.5	No effect	SFA ≥ 100 VFA ≥ 100 L/S ratio < 1	No effect
Tahtaci 2023 [33]	SMI 38.9	No effect	*Not specified.*	Worsened prognosis
Trestini 2023 [34]	SMI < 41	No effect	VAT gain > 10%	Worsened prognosis

L/S ratio, liver/spleen ratio (cm^2^/m^2^); PsoMA, psoas muscle area (mm^2^); SFA, subcutaneous fat area (cm^2^); SMI, skeletal muscle index (cm^2^/m^2^); VAT, visceral adipose tissue (cm^2^); VFA, visceral fat area (cm^2^); V/S ratio, visceral adipose tissue to subcutaneous adipose tissue ratio

### Pathological response

Sixteen studies assessed pathological response and demonstrated variable findings. A minority of studies reported statistically significant associations between sarcopenia and reduced response [18, 23, 29], while several studies reported no significant association. Among these, three studies reported significant associations between sarcopenia and poorer pathological response [18, 23, 29]. Some studies reported statistically significant associations between sarcopenia and pathological response. For example, Guo [23] reported a negative correlation between sarcopenia and both Miller–Payne grade and the achievement of pathological response. Lee [29] reported that reductions in skeletal muscle mass before or after NAC were associated with poorer pathological response. Similarly, Amitani [18] reported that patients with increases in skeletal muscle index (SMI) during NAC had higher rates of pathological response compared with those with stable or decreased SMI.

Findings regarding adiposity and pathological response were inconsistent across studies. One study reported an association between higher visceral fat area (VFA) and liver-to-spleen (L/S) ratio and reduced pathological response [31], whereas another study reported improved response associated with higher visceral adiposity measures, including visceral adipose tissue (VAT) and the VAT/SAT ratio [22]. The remaining eleven studies did not identify a significant association between sarcopenia or adiposity and pathological response [19, 21, 24–26, 28, 30, 32–34, 36]. However, one study reported that skeletal muscle density (SMD), rather than skeletal muscle index (SMI), was associated with achievement of pathological response in patients receiving neoadjuvant chemoimmunotherapy, although this finding was not observed in patients receiving NAC alone [30]. 

### NAC toxicity

Across included studies, the assessment of chemotherapy toxicity was inconsistently reported. Some studies referred to general or hematologic toxicity, while others described treatment-related toxicity without clearly specifying parameters or grading systems. Findings regarding NAC toxicity were variable. Three of the six included studies did not identify a significant association between sarcopenia or adiposity and toxicity outcomes [28, 30, 35]. In contrast, two studies reported associations between both sarcopenia and adiposity and increased toxicity [20, 37]. For example, one study reported associations between multiple skeletal muscle and adiposity measures and hematologic toxicity during NAC [20], while another reported that higher fat mass and relative lean mass were associated with an increased likelihood of toxicity-related treatment modifications [37]. 

One study reported associations between body composition and specific hematologic toxicities, with sarcopenia linked to higher rates of anaemia and adiposity linked to higher rates of neutropenia [27]. Another study reported that skeletal muscle density (SMD) was associated with treatment-related outcomes in patients receiving neoadjuvant chemoimmunotherapy, including relative dose intensity and treatment discontinuation, whereas no such associations were observed with skeletal muscle index (SMI) or in patients receiving NAC alone [30]. 

### Prognosis

Findings regarding prognosis were limited and variable. Only one study reported an association between sarcopenia and poorer survival outcomes [18]. This study described reduced survival among patients with decreases in skeletal muscle index (SMI) during NAC. In contrast, another study reported an association between baseline SMI and overall survival, although the direction of this association differed from other findings [21]. Findings regarding adiposity and prognosis were variable across studies. Six studies reported associations between adiposity measures and survival outcomes [19, 21, 25, 26, 33, 34]. Several studies described associations between increases in visceral adipose tissue during NAC and poorer disease-free survival [19, 34], while others reported associations between baseline visceral fat measures and reduced survival outcomes [25, 26, 33]. 

However, the direction and consistency of these associations varied across studies, and not all studies observed differences in overall survival across body composition parameters [26]. Neither sarcopenia nor adiposity was associated with prognosis in three studies [28, 30, 31]. However, one study reported that skeletal muscle density (SMD) was associated with improved survival outcomes, including event-free, progression-free, and overall survival, in patients receiving neoadjuvant chemoimmunotherapy, although these findings were not observed in patients receiving NAC alone [30]. Another study reported no association between sarcopenia and survival but identified an association between sarcopenic obesity and overall survival [33]. Overall, there was considerable heterogeneity across studies in both body composition assessment methods and reported clinical outcomes.

## Discussion

In this scoping review of literature published between 2010 and 2025, twenty studies were identified that examined the relationship between body composition parameters and outcomes including pathological response, chemotherapy toxicity, and prognosis following NAC in breast cancer patients. Substantial heterogeneity was observed across studies in study design, body composition assessment methods, and outcome definitions. Overall, findings were inconsistent, with variability in measurement techniques and reported outcomes limiting comparability and the ability to draw consistent conclusions. Only one of the included studies was prospective in design [37], and most studies focused on pathological response, with fewer examining chemotherapy toxicity and prognosis. While CT was the most commonly used modality for assessing body composition, alternative techniques such as MRI and BIA have emerged in more recent studies [11, 23, 32]. Similarly, although assessments were most frequently performed at the L3 level, variation in anatomical measurement sites (e.g., L1 and T4) and alternative muscle metrics (e.g., psoas muscle area) were observed [20, 28, 29, 32]. The increasing use of these approaches, alongside the predominance of studies published since 2020, likely reflects growing interest in body composition assessment in breast cancer.

Findings regarding pathological response were variable, with a greater number of studies reporting no significant association with body composition than those reporting associations. A small number of studies reported associations between skeletal muscle measures and pathological response, although the timing and definition of these measures differed across studies. Findings for adiposity were similarly inconsistent, with some studies reporting reduced response associated with higher visceral adiposity measures, while others reported improved response. This variability may reflect differences in adiposity metrics and definitions, including variation in liver-to-spleen ratio cut-offs and other parameters, which limits comparability across studies.

For chemotherapy toxicity, approximately half of the included studies reported associations with body composition measures. However, considerable variation was observed in both body composition parameters (e.g., fat mass vs. skeletal muscle index) and toxicity outcomes (e.g., treatment modifications vs. hematologic toxicity), limiting generalisability. Differences in patient populations, tumour subtypes, and methods of adiposity assessment may also contribute to inconsistent findings. Evidence regarding prognosis was limited and variable. Only one study reported an association between sarcopenia and poorer survival outcomes, while findings for adiposity were more frequently reported but remained inconsistent across studies. Some studies suggested associations between visceral adiposity measures and specific survival outcomes, although the direction and magnitude of these associations varied. Differences in measurement timing and study design further limit comparability.

Overall, these findings highlight a lack of concordance in study design, measurement approaches, and reporting across the literature. This is consistent with previous work [44], which identified heterogeneity in body composition research as a key limitation to evidence synthesis. The absence of standardised definitions and methodologies presents a significant barrier to interpretation and clinical application. Given the limited number of studies, particularly in relation to chemotherapy toxicity, further research is required. Prospective studies using standardised body composition measures and outcome definitions would be valuable in improving the understanding of the relationship between body composition and NAC outcomes in breast cancer.

### Limitations

This review has several limitations. First, screening and study selection were performed by a single reviewer, which may increase the risk of selection bias. While all outcomes were extracted, the narrative synthesis may have emphasised statistically significant associations, which may underrepresent studies reporting no association and should be interpreted with caution. There was substantial heterogeneity in body composition assessment methods across studies, including variation in imaging modality, anatomical level of measurement and definitions of sarcopenia and adiposity. This heterogeneity limits comparability between studies and reduces the ability to draw consistent conclusions regarding the relationship between body composition and breast cancer outcomes. Additionally, as this review focused on neoadjuvant chemotherapy settings, extrapolation of findings to adjuvant chemotherapy contexts may be limited. These findings also highlight important gaps in the literature, including a lack of standardised definitions of body composition and inconsistent outcome reporting.

## Conclusion

Research into body composition in breast cancer has increased in recent years. However, evidence regarding its relationship with neoadjuvant chemotherapy outcomes remains heterogeneous and inconsistent. Further standardised studies are needed to clarify these associations.

## Supplementary Information

Below is the link to the electronic supplementary material.

Supplementary Material 1

## Data Availability

No datasets were generated or analysed during the current study.

## References

[1] Kim J, Harper A, McCormack V et al (2025) Global patterns and trends in breast cancer incidence and mortality across 185 countries. Nat Med 31:1154–1162. 10.1038/s41591-025-03502-3 39994475 10.1038/s41591-025-03502-3

[2] Organisation WH (2024) Breast cancer. https://www.who.int/news-room/fact-sheets/detail/breast-cancer

[3] Huang J, Chan PS, Lok V, Chen X, Ding H, Jin Y et al (2021) Global incidence and mortality of breast cancer: A trend analysis. Aging 13(4):5748. 10.18632/aging.202502 33592581 10.18632/aging.202502 PMC7950292

[4] Aleixo GFP, Shachar SS, Deal AM et al (2020) The association of body composition parameters and adverse events in women receiving chemotherapy for early breast cancer. Breast Cancer Res Treat 182:631–642. 10.1007/s10549-020-05731-1 32519169 10.1007/s10549-020-05731-1

[5] Ceniccola GD, Castro MG, Piovacari SMF et al (2019) Current technologies in body composition assessment: advantages and disadvantages. Nutrition 62(20181206):25–31. 10.1016/j.nut.2018.11.028 30826596 10.1016/j.nut.2018.11.028

[6] Iwase T, Wang X, Shrimanker TV et al (2021) Body composition and breast cancer risk and treatment: mechanisms and impact. Breast Cancer Res Treat 186:273–283. 10.1007/s10549-020-06092-5 33475878 10.1007/s10549-020-06092-5

[7] Chan DSM, Vieira AR, Aune D et al (2014) Body mass index and survival in women with breast cancer—systematic literature review and meta-analysis of 82 follow-up studies. Ann Oncol 25:1901–1914. 10.1093/annonc/mdu042 24769692 10.1093/annonc/mdu042 PMC4176449

[8] Greenlee H, Unger JM, Leblanc M et al (2017) Association between Body Mass Index and Cancer Survival in a Pooled Analysis of 22 Clinical Trials. Cancer Epidemiol Biomarkers Prev 26:21–29. 10.1158/1055-9965.epi-15-1336 27986655 10.1158/1055-9965.EPI-15-1336 PMC5370550

[9] Kwan ML, Chen WY, Kroenke CH et al (2012) Pre-diagnosis body mass index and survival after breast cancer in the After Breast Cancer Pooling Project. Breast Cancer Res Treat 132:729–739. 10.1007/s10549-011-1914-3 22187127 10.1007/s10549-011-1914-3 PMC3507508

[10] Caan BJ, Cespedes Feliciano EM, Prado CM et al (2018) Association of Muscle and Adiposity Measured by Computed Tomography With Survival in Patients With Nonmetastatic Breast Cancer. JAMA Oncol 4:798. 10.1001/jamaoncol.2018.0137 29621380 10.1001/jamaoncol.2018.0137 PMC6584322

[11] Danesi V, Andalò A, Cavallucci M et al (2024) Body weight and body surface area of adult patients with selected cancers: An Italian multicenter study. PLoS ONE 19(12):e0314452 Published 2024 Dec 17. 10.1371/journal.pone.0314452 39689072 10.1371/journal.pone.0314452 PMC11651557

[12] Aleixo GFP, Valente SA, Wei W et al (2023) Sarcopenia detected with bioelectrical impedance versus CT scan and chemotherapy tolerance in patients with early breast cancer. Breast Cancer 30:101–109. 10.1007/s12282-022-01401-w 36063308 10.1007/s12282-022-01401-w

[13] Shachar SS, Deal AM, Weinberg M et al (2017) Body Composition as a Predictor of Toxicity in Patients Receiving Anthracycline and Taxane–Based Chemotherapy for Early-Stage Breast Cancer. Clin Cancer Res 23:3537–3543. 10.1158/1078-0432.ccr-16-2266 28143874 10.1158/1078-0432.CCR-16-2266 PMC5511549

[14] Molfino A, Imbimbo G, Pisegna S et al (2025) Impact of Body Composition Changes on Treatment-Related Toxicities and Clinical Outcomes in HER2-Positive Metastatic Breast Cancer Patients Receiving Trastuzumab Deruxtecan. Cancers (Basel) 17:20250919. 10.3390/cancers17183063 10.3390/cancers17183063 PMC12468189 41008905

[15] Wong AL, Seng KY, Ong EM et al (2014) Body fat composition impacts the hematologic toxicities and pharmacokinetics of doxorubicin in Asian breast cancer patients. Breast Cancer Res Treat 144:143–152. 10.1007/s10549-014-2843-8 24481679 10.1007/s10549-014-2843-8

[16] Tricco AC, Lillie E, Zarin W et al (2018) PRISMA Extension for Scoping Reviews (PRISMA-ScR): Checklist and Explanation. Ann Intern Med 169:467–473. 10.7326/m18-0850 30178033 10.7326/M18-0850

[17] Arksey H, O’Malley L (2005) Scoping studies: towards a methodological framework. Int J Soc Res Methodol 8:19–32. 10.1080/1364557032000119616

[18] Amitani M, Oba T, Kiyosawa N et al (2022) Skeletal muscle loss during neoadjuvant chemotherapy predicts poor prognosis in patients with breast cancer. BMC Cancer 22:327. 10.1186/s12885-022-09443-1 35346102 10.1186/s12885-022-09443-1 PMC8962250

[19] Carbognin L, Trestini I, Sperduti I et al (2020) Prognostic impact of body composition (BC) changes during neoadjuvant chemotherapy (NACT) in breast cancer patients (pts). Ann Oncol 31:S314

[20] Chen M, Wang P, Li Y et al (2024) Prediction of hematologic toxicity in luminal type breast cancer patients receiving neoadjuvant chemotherapy using CT L1 level skeletal muscle index. Sci Rep 14:8604. 10.1038/s41598-024-58433-9 38615057 10.1038/s41598-024-58433-9 PMC11016056

[21] Del Fabbro E, Parsons H, Warneke CL et al (2012) The relationship between body composition and response to neoadjuvant chemotherapy in women with operable breast cancer. Oncologist 17:1240–1245. 10.1634/theoncologist.2012-0169 22903527 10.1634/theoncologist.2012-0169 PMC3481889

[22] Dulgar O, Ibisoglu EO, Ay S et al (2023) Is adipose tissue metabolic activity a predictor of pathological responses to neoadjuvant treatment in breast cancer. Revista Esp de Med nuclear e imagen Mol 42:10–15. 10.1016/j.remnie.2022.08.003 10.1016/j.remnie.2022.08.003 35988844

[23] Guo J, Meng W, Li Q et al (2024) Pretreatment Sarcopenia and MRI-Based Radiomics to Predict the Response of Neoadjuvant Chemotherapy in Triple-Negative Breast Cancer. Bioengineering 11:663 39061745 10.3390/bioengineering11070663 PMC11274092

[24] Isıklar A, Yilmaz E, Basaran G (2024) The Relationship Between Body Composition and Pathological Response to Neoadjuvant Chemotherapy in Breast Cancer Patients. Cureus. ; 16 10.7759/cureus.61145 PMC11199927 38933645

[25] Iwase T, Parikh A, Dibaj SS et al (2021) The prognostic impact of body composition for locally advanced breast cancer patients who received neoadjuvant chemotherapy. Cancers 13:608 33557032 10.3390/cancers13040608 PMC7913702

[26] Iwase T, Sangai T, Nagashima T et al (2016) Impact of body fat distribution on neoadjuvant chemotherapy outcomes in advanced breast cancer patients. Cancer Med 5:41–48. 10.1002/cam4.571 26626021 10.1002/cam4.571 PMC4708907

[27] Jang MK, Park S, Park C et al (2023) Hematologic toxicities, sarcopenia, and body composition change in breast cancer patients undergoing neoadjuvant chemotherapy. Support Care Cancer 31:419. 10.1007/s00520-023-07890-5 37354335 10.1007/s00520-023-07890-5

[28] Karaca M, Alemdar MS, Deniz Karaca O et al (2024) Sarcopenia’s Role in Neoadjuvant Chemotherapy Outcomes for Locally Advanced Breast Cancer: A Retrospective Analysis. Med Sci Monit Int Med J Exp Clin Res 30:e945240. 10.12659/MSM.945240 10.12659/MSM.945240 PMC11608060 39587458

[29] Lee BM, Cho Y, Kim JW et al (2021) Association between skeletal muscle loss and the response to neoadjuvant chemotherapy for breast cancer. Cancers 13:1806 33918977 10.3390/cancers13081806 PMC8070318

[30] Mun HS, Kim SH, Lee J et al (2025) Skeletal Muscle Density as a Predictive Marker for Pathologic Complete Response in Triple-Negative Breast Cancer Treated with Neoadjuvant Chemoimmunotherapy. Cancers 17:1768 40507249 10.3390/cancers17111768 PMC12153542

[31] Omarini C, Palumbo P, Pecchi A et al (2019) Predictive role of body composition parameters in operable breast cancer patients treated with neoadjuvant chemotherapy. Cancer Manage Res 2019: 9563–9569 10.2147/CMAR.S216034 PMC6859164 32009814

[32] Rossi F, Lambertini M, Brunetti N et al (2023) Muscle mass loss in breast cancer patients of reproductive age (≤ 45 years) undergoing neoadjuvant chemotherapy. Radiol Med 128:49–57. 10.1007/s11547-022-01574-6 36536266 10.1007/s11547-022-01574-6

[33] Tahtaci G, Yildiz B, Aydos U (2023) 368P Impact of PET-CT-determined sarcopenia on survival and pathological complete response in breast cancer patients with neoadjuvant chemotherapy. Ann Oncol 34:S329–S330

[34] Trestini I, Caldart A, Cintoni M et al (2023) Predictive and prognostic effect of computed tomography-derived body composition analysis during neoadjuvant chemotherapy for operable and locally advanced breast cancer. Nutrition (Burbank, Los Angeles County. Calif) 105:111858. 10.1016/j.nut.2022.111858 10.1016/j.nut.2022.111858 36323147

[35] Di Leone A, Filippone A, Maggiore C et al (2025) The role of body composition in neurological and hematologic toxicity in a retrospective analysis of 120 breast cancer patients undergoing neoadjuvant chemotherapy: the COMBOTOX study. Breast Cancer Res Treat 210:205–213. 10.1007/s10549-024-07553-x 39630164 10.1007/s10549-024-07553-x PMC11787179

[36] Yirgin IK, Has D, Arslan G et al (2020) Comparison between body composition parameters and response to neoadjuvant chemotherapy by using pre-treatment PET CT in locally advanced breast cancer. Eur J Radiol Open 7:100286 33294497 10.1016/j.ejro.2020.100286 PMC7689395

[37] n Den Berg MMGA, Kok DE, Posthuma L et al (2019) Body composition is associated with risk of toxicity-induced modifications of treatment in women with stage I–IIIB breast cancer receiving chemotherapy. Breast Cancer Res Treat 173:475–481. 10.1007/s10549-018-5014-5 30353244 10.1007/s10549-018-5014-5 PMC6394786

[38] Aleixo GFP, Williams GR, Nyrop KA et al (2019) Muscle composition and outcomes in patients with breast cancer: meta-analysis and systematic review. Breast Cancer Res Treat 177:569–579. 10.1007/s10549-019-05352-3 31292800 10.1007/s10549-019-05352-3

[39] Cespedes Feliciano E, Chen WY (2018) Clinical implications of low skeletal muscle mass in early-stage breast and colorectal cancer. Proc Nutr Soc 77:382–387. 10.1017/s0029665118000423 29860952 10.1017/S0029665118000423 PMC6197885

[40] Litton JK, Gonzalez-Angulo AM, Warneke CL et al (2008) Relationship Between Obesity and Pathologic Response to Neoadjuvant Chemotherapy Among Women With Operable Breast Cancer. J Clin Oncol 26:4072–4077. 10.1200/jco.2007.14.4527 18757321 10.1200/JCO.2007.14.4527 PMC6557586

[41] Mak S, Thomas A (2022) Steps for Conducting a Scoping Review. J Graduate Med Educ 14:565–567. 10.4300/jgme-d-22-00621.1 10.4300/JGME-D-22-00621.1 PMC9580325 36274762

[42] Rasmy A, Sorour Y (2020) Effect of Obesity on Neoadjuvant Systemic Therapy Outcomes in Patients with Early Breast Cancer: A Retrospective Institutional Study. Asian Pac J Cancer Prev 21:683–691. 10.31557/apjcp.2020.21.3.683 32212794 10.31557/APJCP.2020.21.3.683 PMC7437318

[43] Wang H, Yee D, Potter D et al (2024) Impact of body mass index on pathological response after neoadjuvant chemotherapy: results from the I-SPY 2 trial. Breast Cancer Res Treat 204:589–597. 10.1007/s10549-023-07214-5 38216819 10.1007/s10549-023-07214-5 PMC10959799

[44] Klassen PN, Mazurak VC, Thorlakson J et al (2023) Call for standardization in assessment and reporting of muscle and adipose change using computed tomography analysis in oncology: A scoping review. J Cachexia Sarcopenia Muscle 14:1918–1931. 10.1002/jcsm.13318 37675809 10.1002/jcsm.13318 PMC10570077

