# Supplementary material for: Associations of body composition with neoadjuvant chemotherapy response, toxicity, and prognosis in breast cancer: a scoping review
Source: Cancer Chemother Pharmacol. 2026 Aug 12;96(1):89. doi: 10.1007/s00280-026-04940-7 (PMC13461859; doi:10.1007/s00280-026-04940-7)
Supplement: Supplementary file 1 [file 280_2026_4940_MOESM1_ESM.docx]

Appendix 1: Search strategies

**Google scholar**

allintitle: neoadjuvant "body composition" OR "muscle mass" OR "adipos *" OR sarcopenia OR "skeletal muscle" "breast cancer"

**PubMed**

"Breast Neoplasms"[MeSH] AND "Neoadjuvant Therapy"[MeSH] AND ("body composition" OR "muscle mass" OR "adipos*" OR "sarcopenia" or "skeletal muscle") AND (“survival” OR “prognosis” OR “toxic*” OR “response”)

**Medline**

"Breast Neoplasms/” AND "Neoadjuvant Therapy/" AND (Muscle, Skeletal/ OR Body Composition/ OR Sarcopenia/ OR Adiposity/ OR Adipose Tissue/)

**Embase**

“breast cancer”/ AND “neoadjuvant chemotherapy”/ AND (“body composition” OR “sarcopenia”/ OR “skeletal muscle”/ or “muscle mass”/ OR “adipose tissue”/) AND (“pathological response”/ OR “cancer prognosis”/ OR chemotoxic* OR chemotherapy toxic* OR toxic*)

Appendix 2: Data extraction template.

**General information**

Study ID

Title (Title of paper / abstract / report that data are extracted from)

Lead author

Country in which the study conducted

1. United States
2. UK
3. Canada
4. Australia
5. Other

Year published

Notes

**Characteristics of included studies**

**Methods**

Aim of study

Study design

1. Randomised controlled trial
2. Non-randomised experimental study
3. Retrospective cohort study
4. Prospective cohort study
5. Cross sectional study
6. Case control study
7. Qualitative research
8. Prevalence study
9. Case series
10. Case report
11. Other

Primary outcome investigated

1. Toxicity
2. pCR
3. Prognosis
4. Other

Prognosis measure used:

1. Overall survival (OS)
2. Disease free survival (DFS)
3. Other

Toxicity measure used

Outcome measure definition

Secondary outcome investigated

Assessment timing

Assessment method

1. CT
2. CT L3
3. CT L1
4. BIA
5. DEXA
6. MRI
7. Other

Component assessed

1. Skeletal muscle area (SMA)
2. Skeletal muscle index (SMI)
3. Skeletal muscle mass (SMM)
4. Psoas muscle area (PMA)
5. Intramuscular adipose tissue (IMAT)
6. Subcutaneous adipose tissue (SAT)
7. Subcutaneous fat area (SFA)
8. Visceral adipose tissue (VAT)
9. Visceral fat area (VFA)
10. Other

Component definitions / cut-offs

Adiposity assessment method

**Participants**

Population description

Start date

End date

Inclusion criteria

Exclusion criteria

Total number of participants

Cancer stage

Breast cancer histological type

Breast cancer subtype

NAC Regimen

Follow up time

**Results**

Age

Outcome 1 findings

Outcome 2 findings

Outcome 3 findings

Additional findings of note

Study conclusions

Study limitations

Appendix 3: Further data extracted – participant characteristics.

Table A: Participant characteristics of included studies.

| **Study ID** | **Population description** | **Eligibility criteria** | **Age (yrs)** | **Cancer stage** | **Breast cancer histological type** | **Breast cancer subtype groups** | **NAC Regimen** |
| --- | --- | --- | --- | --- | --- | --- | --- |
| Amitani 2022^18^ | Patients with BC and a performance status of ECOG 0, who underwent NAC and subsequent surgery at Shinshu University Hospital between February 2010 and December 2017. | Inclusion criteria:  i) patients with pathologically confirmed BC by core needle biopsy  ii) patients who underwent CT or PET-CT within both 4 weeks of initiation of NAC and surgery following NAC.  Exclusion criteria:  Patients who could not complete NAC owing to chemotoxicity or disease progression during NAC. | 52.3 (mean ± 10.1 SD) | II - III | IDC Special type | Luminal  Luminal-HER2  HER2-enriched  TN | - 4 cycles EC/FEC: tri-weekly anthracycline-based FEC (500mg/m2 fluorouracil, 100mg/m2 epirubicin, and 500mg/m2 cyclophosphamide) regimen *[except for one patient who had been treated with EC regimen (60mg/m2 epirubicin and 600mg/m2 cyclophosphamide) in another hospital and was transferred to study hospital for surgery]* - Followed by 4 cycles of DTX or PTX: taxane regimens, including triweekly DTX 75mg/m2 or weekly paclitaxel (PTX) 80mg/m2.  - In HER2+ patients - 6mg/kg (triweekly) or 2mg/kg  (weekly) TRA simultaneously administered with taxane regimen. |
| Carbognin 2020^19^ | BC patients treated with NAC at Oncology of Verona between 2014 and 2019. | *Not specified.* | 47 (median; range 30-72) | *“operable/locally-advanced breast cancer”* | *Not specified.* | *Not specified.* | *Not specified.* |
| Chen 2024^20^ | Patients diagnosed with primary BC who underwent NAC followed by surgical intervention at the First Hospital of Lanzhou University between July 2018 and September 2023. | Inclusion criteria:  (1) luminal-type BC pathology;  (2) unilateral breast cancer in females;  (3) completion of 4-8 cycles of NAC;  (4) underwent chest and/or abdominal CT examinations before NAC.  Exclusion criteria:  (1) anti-tumour therapy administration before diagnosis;  (2) pregnancy or lactation;  (3) coexistence of other malignancies from different tissue origins;  (4) missing essential data. | 49.4 (mean; range 25-74) | *Not specified.* |  | Luminal-type | *Not specified.* |
| Del Fabbro 2012^21^ | The Breast Cancer Management System database of MD Anderson Cancer Center (MDACC) was used to identify women with nonmetastatic, primary, invasive ductal or lobular noninflammatory BC treated with NAC prior to being eligible for surgical treatment at MDACC, between December 2000 and December 2004. | Inclusion criteria:  All patients included in the study had nonmetastatic BC treated with NAC and CT scans prior to chemotherapy with axial slices over the third lumbar vertebra (L3). Primary invasive ductal or lobular noninflammatory BC was diagnosed using core needle biopsy, and tissue was evaluated using pathology before the initiation of NAC.  Exclusion criteria:  Patients who had partial or definitive surgery before receiving NAC, were pregnant, refused surgery after NAC, or had a pathological response assessment 1 year after NAC. | *Not specified.* | I - III | Primary IDC  Lobular noninflammatory | ER- ER+ PR- PR+ HER2- HER2+ | *Not specified* |
| Di Leone 2025^35^ | Patients who underwent NAC for non-metastatic BC at Policlinico Universitario Agostino Gemelli IRCCS in Rome (Italy) between May 2018 and December 2020. |  | 49.3 (mean; SD 11.9) | *“non-metastatic breast cancer”* |  | Luminal B/ HR+ /HER2-  Luminal B/ HR+/ HER2+  Non-luminal/ HER2+  TN | i) Anthracycline-taxane-based protocols  ii) Anthracycline-taxanes plus carboplatin |
| Dulgar 2023^22^ | Patients diagnosed with stage II to III BC between the years 2016 and 2020 who had a PET-CT performed prior to NAC.  Seventy-four patients had also PET-CT after completion of chemotherapy. |  | 51 (median; range 26-78) | II - III |  | HR+ HR- HER2+  HER2- | - 4 cycles anthracycline regimen (doxorubicin 60 mg/m2 plus cyclophosphamide 600 mg/m2, on day 1, every 21 days)  - Followed by weekly PTX dose of 80 mg/m2 for 12 weeks  For HER2+ (in addition to chemotherapy)  - TRA 8 mg/kg loading, and 6 mg/kg maintenance dose, repeated every 3 weeks - PER 840 mg loading dose given on day 1, and maintenance dose with 420 mg in every 3 weeks.  - After surgery, TRA continued for 1 year. |
| Guo 2024^23^ | Female patients with TNBC who underwent NAC treatment followed by surgery between January 2012 and September 2021 in study hospital. | Inclusion criteria:  (i) patients who had primary TNBC confirmed by biopsy and without distant metastasis;  (ii) patients who underwent complete NAC therapy;  (iii) patients who underwent surgery at study hospital after completion of NAC, with postoperative pathological examination including Miller-Payne grading;  (iv) breast MRI performed before treatment, including T1C sequence;  (v) pre-treatment CT scan was performed, covering the level of L3 vertebra.  Exclusion criteria:  (i) patients who underwent biopsy at an external institution or were unable to obtain pretreatment pathological results;  (ii) patients who did not complete NAC or received non-standard treatments;  (iii) patients who underwent surgery at an external institution or were unable to obtain Miller-Payne grading;  (iv) MRI and CT quality was insufficient to obtain measurable results. | 48.7 (mean) | T1-4; N0-3; M0 |  | TN | Regimens were based on anthracyclines and taxanes +/-alkylating agents.  For some high-risk patients, platinum-containing chemotherapy regimens were administered. |
| Isıklar 2024^24^ | Individuals with early-stage BC who underwent NAC between February 2017 and December 2022. | Participants chosen based on having had positron emission tomography-computed  tomography (PET-CT) or chest/abdominal CT scans at time of cancer diagnosis. | 46 (median; range = 29-72) | *Early stage & locally advanced* |  | HR+ HER2+ TN | *Not specified.* |
| Iwase 2016^26^ | Locally advanced BC patients who underwent surgery after NAC between January 2004 and December 2012. | Inclusion criteria:  1) ECOG performance status of 0-1 2) A clinical T2 classification or higher (tumor size ≥ 20 mm) based on the American Joint Committee on Cancer/Union for International Cancer Control TNM classification  3) A positive axillary lymph node proven by fine needle aspiration cytology.  Exclusion criteria:  Patients with distant metastasis, identified based on CT and 99mtechnetium bone scans performed before NAC, were excluded from the study. | 54 (median; range = 30-76) | II - III | Ductal Other | ER+/ HER2-  ER+/ HER2+  ER-/ HER2+  TN | Anthracycline, followed by taxane.  HER2+ cases - TRA for 1 year. |
| Iwase 2021^25^ | Patients with BC who underwent NAC between January 2007 and June 2015 at The University of Texas MD Anderson Cancer Centre. | Inclusion criteria:  (1) pathologically confirmed stage III invasive BC per American Joint Committee on Cancer diagnostic criteria; (2) underwent multi-detector computed tomography with images spanning from the top of the diaphragm to the umbilicus level before initiating NAC;  (3) thickness of the CT slices was at least 5 mm;  (4) sufficient clinical and pathologic information and follow-up data for analysis | 49 (median) | III |  | ER+/HER2- ER+/HER2+ ER-/HER2+ TN | *Not specified* |
| Jang 2023^27^ | Data provided by the Big Data Team of Yonsei University Health Systems in South Korea and included BC patients with abdominal CT images taken before and after NAC between January 2017 and November 2020. | Inclusion criteria:  Women aged 20 years or older who had completed NAC, received two abdominal CT scans (at the start of chemotherapy and after chemotherapy), and completed surgery at least 6 months prior to data collection. | 53.29 ± 9.68 (mean ± SD; range = 26-78) | I - III |  | HR+/HER2-  HR+/ HER2+  HR-/HER2+  TN | i) Anthracycline and cyclophosphamide followed by taxane every 3 weeks  ii) Anthracycline and cyclophosphamide followed by taxane weekly  iii) DTX, carboplatin, TRA, and PER |
| Karaca 2024^28^ | Patients diagnosed with invasive BC who received NAC at the Health Sciences University Antalya Training and Research Hospital between 2015 and 2021. | Inclusion criteria:  Patients with histologically confirmed stage II or III BC, who underwent surgery after NAC and had complete follow-up data.  Exclusion criteria:  Early-stage or metastatic BC cases and those with incomplete data. | 50.0 (mean ± 11.5 SD) | II -III |  | HER2+  TN  Luminal A Luminal B | Anthracycline-cyclophosphamide followed by PTX or DTX.  TRA/PER added for HER2+ cases. |
| Lee 2021^29^ | Patients diagnosed with BC who were treated with NAC, surgery, and radiotherapy from March 2013 to September 2019. | Inclusion criteria:  i) age more than 18 years ii) treatment with either partial mastectomy or total mastectomy  iii) treatment with NAC and adjuvant RT iv) available medical records  Exclusion criteria:  i) follow-up loss ii) inability to measure the skeletal muscle mass iii) synchronous malignancies | 48 (median) | I - III |  | ER+ ER- PR+ PR- HER2+  HER2- | i) Adriamycin based ii) Adriamycin and Taxol based iii) Taxol based iv) Trastuzumab based +/- Selective estrogen receptor modulator +/- Aromatase inhibitor |
| Mun 2025^30^ | TNBC patients who underwent NAC at Seoul St. Mary's Hospital, Catholic University of Korea, from March 2021 to March 2024, via receiving paclitaxel/carboplatin followed by doxorubicin/cyclophosphamide, with or without pembrolizumab. | Inclusion criteria:  1) histologically confirmed TNBC (2) stage II-III disease;  (3) neoadjuvant treatment consisting of paclitaxel/carboplatin followed by doxorubicin/cyclophosphamide, with or without pembrolizumab | 53 (median) | II - III |  | TN | - 4 cycles weekly PTX (80 mg/m2) combined with three-weekly carboplatin (AUC 5)  - Followed by 4 cycles of three-weekly doxorubicin (60 mg/m2) and cyclophosphamide (600 mg/m2) - In NACI group, up to eight cycles of three-weekly pembrolizumab given |
| Omarini 2019^31^ | Patients treated with NAC for early BC in Modena Cancer Centre between 2005 and 2017. | Inclusion criteria:  Histologically proven diagnosis of BC (stage I-III) treated with primary chemotherapy either within clinical trials or on the basis of standard guidelines.  Weight and height available and/or pre-treatment CT scan images. | 50 (median; range 25-80) | I - III |  | HR+ HER2+ TN | i) Anthracycline-taxane-based  ii) Taxane-based  iii) Anthracycline-based  iv) Platinum-based |
| Rossi 2023^32^ | Patients of reproductive age (<45 years), who have undergone different  regimes of NAC for histologically proven primary BC, whose clinical and radiological response was evaluated in the University Hospital from January 2019 to September 2021.  Patients included were enrolled in the existing coordinating centre of the PREgnancy and Fertility (PREFER) study (NCT02895165). | Inclusion criteria:  i) patients of reproductive age (≤45 years) who had histologically confirmed BC by core needle biopsy and were enrolled in the PREFER study;  ii) those who underwent MRI before and after NAC at study institution.  Exclusion criteria:  i) tumour involving and invading the pectoralis muscle  ii) unknown immunohistochemical markers  iii) MRI exams before or after NAC not retrievable  iv) inaccurate images due to artifacts (e.g., susceptibility artifacts due to infusion port) or previous breast surgery (e.g., breast implants) v) previous systemic, surgical or radiotherapy treatment (e.g., due to contralateral BC) that may have affected body composition. | 37 (mean; SD ±4.96) | *Not specified.* | IDC  Carcinosarcoma | Luminal disease HER2+ TN | One of the following:  i) 4 EC cycles and weekly PTX for 8-12 weeks  ii) 4 EC cycles followed by 8-12 weeks of carboplatin and PTX  iii) 4 EC cycles followed by 8-12 weeks of PTX and TRA  iv) 8-12 weeks of PTX and carboplatin |
| Tahtaci 2023^33^ | BC patients treated with NAC between 2014 and 2021. |  | *Not specified.* | *Not specified.* |  |  | *Not specified.* |
| Trestini 2023^34^ | Patients with BC treated with NAC at the Oncology Unit of the University of Verona, Azienda Ospedaliera Universitaria Integrata of Verona, between January 2014 and December 2019. | Inclusion criteria:  Histologically proven diagnosis of BC with clinical stage I-III; Anthracyclines and/or taxane-based NAC; Surgery for primary tumour at the completion of NAC; Complete data on weight and BMI before and after NAC; Availability of pre- and post-treatment CT scan images, provided by PET-CT staging.  Exclusion criteria:  Patients who presented significant nutritional impact symptoms and/or who received nutrition support were excluded. | 47 (median; range 30-72) | I - III |  | - Luminal A (HR+/ HER2-)  - Luminal B (HR+)/ HER2-  - Luminal B (HR+)/ HER2+  - HER2-enriched  - TN | i) Anthracycline-taxane-based  ii) Taxane-based |
| van den Berg 2019^37^ | Patients were part of the COBRA-study, an observational multi-centre study among BC patients receiving NAC. Eligible patients were recruited by the medical staff from 11 academic and peripheral hospitals in the Netherlands prior to commencement of NAC between May 2013 and September 2016. | Inclusion criteria:  i) Newly diagnosed with operable stage I-IIIB BC and scheduled to receive NAC.  ii) At least 18 years old  iii) Be able to communicate in Dutch.  Exclusion criteria:  i) History of cancer  ii) Previous treatment with chemotherapy  iii) Pregnancy during study period  iv) Dementia or other mental conditions that may affect study procedure compliance. | 51.8 (median) | I - IIIB |  | *Not specified.* | Combined regimes: scheme components administered together during all cycles  - TAC  - FEC  - DTX-cyclophosphamide  - CDT +/- PER  - PT  - CTP Sequential regimes: scheme components administered in different cycles  - AC/PTX +/- TRA  - FEC/DTX  - AC/DTX +/- TRA |
| Yirgin 2020^36^ | Locally advanced BC patients who underwent surgical resection after NAC in a singular hospital between January 2015 and October 2019. | Inclusion criteria:  Patients with locally advanced BC who had  i) PET- CT scan performed for staging  ii) Clinicopathological data, including age, tumour characteristics and treatment with operation history, in the medical records of study institution iii) Core needle biopsy specimens obtained before NAC. | 49 (median; range 21-73) | *Locally advanced breast cancer.* | IDC Invasive lobular Mixed Micropapillary Metaplastic  Mucinous | ER+ ER- PR+ PR- HER2+  HER2-  HER2 suspected  TN Non-TN | *Not specified.* |

AC, doxorubicin-cyclophosphamide; BC, breast cancer; CDT, carboplatin-docetaxel-trastuzumab; CT, computed tomography; CTP, carboplatin-paclitaxel-trastuzumab; DTX, docetaxel; EC, epirubicin-cyclophosphamide; ER+, oestrogen receptor positive; ER-, oestrogen receptor negative; FEC, fluorouracil-epirubicin-cyclophosphamide; HER2+, human epidermal growth factor positive; HER2-, human epidermal growth factor negative; HR+, hormone receptor positive; HR-, hormone receptor negative; IDC, invasive ductal carcinoma; MRI, magnetic resonance imaging; NAC, neoadjuvant chemotherapy; NACI, neoadjuvant chemoimmunotherapy; PET-CT, positron emission tomography–computed tomography; PER, pertuzumab; PR+, progesterone receptor positive; PR-, progesterone receptor negative; PT, paclitaxel-trastuzumab; PTX, paclitaxel; TAC, docetaxel-doxorubicin-cyclophosphamide; TN, triple negative; TNBC; triple negative breast cancer; TRA, trastuzumab.

Appendix 4: Further data extracted – relevant definitions.

Table B: Outcome and component definitions, cut-off values and follow up periods used in included studies.

| **Study ID** | **Relevant outcome definitions** | **Component definitions & cut-offs** |
| --- | --- | --- |
| Amitani 2022^18^ | pCR: no evidence of residual invasive carcinoma in breast tissue, regardless of the axillary lymph node status.  Prognosis:  DFS - time from surgery to the date of detection of locoregional relapse or distant metastases, whichever occurred first.  OS – time from day of surgery to date of death from any cause | % change in SMI = percent change after NAC from that at pretreatment. |
| Carbognin 2020^19^ | *N/A* | *N/A* |
| Chen 2024^20^ | Toxicity: Hematologic toxicity graded according to the radiation therapy oncology group (RTOG) criteria.  Severe hematologic toxicity - grade 3 or higher (e.g. haemoglobin <80 g/L, white blood cells <2.0x10^9^/L, neutrophils <1.0x10^9^/L, platelets <50x10^9^/L).  In cases of multiple grades of anaemia, leukopenia, neutropenia, or thrombocytopenia, the highest grade was considered. | Cut-off value for sarcopenia in women: SMI <39 cm2/m2 at the L3 level.   VFI = VFA divided by square of the height. |
| Del Fabbro 2012^21^ | pCR: No residual invasive carcinoma in either the breast or axillary lymph nodes. Pts with residual ductal carcinoma in situ were included in the pCR group.  Prognosis:  PFS interval - from date of NAC initiation to the date of disease recurrence or metastasis, or, if no recurrence or metastasis was recorded, to the time of last follow-up. | Cut-off value for sarcopenia in women: SMI ≤38.5 cm2/m2 |
| Di Leone 2025^35^ | Toxicity: main chemo-related toxicities in BC patients receiving NAC.  Secondary outcomes: (i) chemotherapy interruption; (ii) common side effects (including hypertransaminasemia, hot flashes, headache, constipation, epigastralgia, onychopathy, osteoarticular pain, asthenia, diarrhea, emesis, and mucositis). |  |
| Dulgar 2023^22^ | pCR: The Miller-Payne grading system used to evaluate pathologic response.  Grade V - complete pathologic response Grade III or IV - non-complete pathologic response  Grade I or II - poor pathologic response Patients grouped into: good pathologic response (MPG score V, IV, III) or poor pathologic response (MPG score I, II). | V/S ratio = VAT SUV divided by SAT SUV. |
| Guo 2024^23^ | pCR: According to NCCN guidelines, and using Miller–Payne (MP) grading: Poor treatment response - MP grade of 1-3 (MP-Low) after NAC  Better treatment response - MP grades 4 and 5 (MP-High)  pCR - MP grade 5 (primary tumour bed without invasive cancer cells but may contain ductal carcinoma in situ) with ypN0 group. Further defined as the absence of residual invasive carcinoma in both the breast and axillary lymph nodes, while carcinoma in situ was permitted in certain cases (ypT0/Tis ypN0). Non-pCR - Patients with residual lesions in the breast or axilla, or both, after NAC. | Cut-off value for sarcopenia in women: SMI < 39 cm2/m2 |
| Isıklar 2024^24^ | pCR: Response to treatment - Residual Cancer Burden (RCB) classification | Cut-off value for sarcopenia in women: SMI ≤ 38.5 |
| Iwase 2016^26^ | pCR: No residual tumor cells in the breast (ypT0) and non-invasive residuals allowed in the intraductal components (ypTis/mic), regardless of the axillary lymph node status (ypN+)  Prognosis: DDFS - time from initial NAC treatment to relapse at any distant site.  Toxicity: NAC dose intensity and relative dose intensity (RDI) calculated using electronically recorded data as follows:  Dose intensity (mg/m2/week) = total dose (mg/m2) / duration of administration (weeks)  RDI = (dose intensity / planned dose intensity) x 100. | SMI = total L3 skeletal muscle mass divided by body surface area (cm2/m2)  Sarcopenic obesity - BMI ≥ 30 and SMI lower than the median value. Liver fat accumulation used as a measure of internal fat distribution. The CT liver-to-spleen attenuation ratio (L/S ratio) used for fatty liver evaluation.  Fatty liver was defined as an L/S ratio of >1.0. Axial cross-sectional CT images taken at the navel level were used for adiposity measurements. |
| Iwase 2021^25^ | pCR: Defined as either an absence of residual tumor (ypT0N0) or non-invasive in situ residual tumour remaining (ypTis/0N0) in the surgical specimen from the primary tumour and axillary lymph nodes. Prognosis: DFS - time from the initial diagnosis to recurrence in distant organs, including bone, liver, lung, or brain.  RFS - time from initial diagnosis to recurrence in any site, including locoregional recurrence or recurrence in the contralateral breast.  OS - time from initial diagnosis to death from any cause. | SMI = SMA divided by body surface area (cm2/m2).  V/S ratio cut off = 34 |
| Jang 2023^27^ | Toxicity: Haematologic toxicities categorized based on Common Terminology Criteria for Adverse Events (CTCAE) version 5.0.  Analysis focused on toxicity grade 3 (severe or medically significant) and grade 4 (life-threatening) adverse events. Eg:  Grade 3 anaemia - haemoglobin < 8.0 g/dL  Grade 3 neutropenia - absolute neutrophil count < 1000 /microL  Grade 3 thrombocytopenia - platelet count < 50,000/microL. | L3 cut-off SMI <38.5 |
| Karaca 2024^28^ | pCR: Absence of invasive cancer in the breast and lymph nodes (ypT0/Tis ypN0).  Prognosis:  DFS – time from surgery to relapse, metastasis, or death. OS – time from diagnosis to death from any cause. | Mean PMA = average of right and left psoas areas Cut-off value for sarcopenia = PMA 415.4 mm^2^ (25^th^ percentile) |
| Lee 2021^29^ | pCR:  Responsiveness to treatment – evaluated using the Response Evaluation Criteria in Solid Tumours.  Responder group = pts showing either complete or partial response  Non-responder group = pts w/ stable or progressive disease | Cut-off value at T4 for skeletal muscle loss group – SMI = 6  SATI = cross-sectional volume of SAT at T4 divided by axial slice thickness. |
| Mun 2025^30^ | Prognosis:  OS - time from start of treatment to death from any cause or the last follow-up date EFS – time from start of NAC to first occurrence of any of the following: disease progression precluding definitive surgery, local or distant recurrence, development of second primary cancer, or death from any cause. | SMD (muscle quality) = mean radiodensity (HU) of the entire cross-sectional muscle area at the L3 level SMI (muscle quantity) = SMA divided by the square of patient's height  NACI group:  SMD categorised into three tertile-based groups (low, medium, high).  SMD tertiles defined with cutoff values at 48 and 53.  Lowest tertile of SMD (<48 HU) indicative of low SMD.  NAC group  SMD cut-off = 44 HU (lowest tertile of SMD) |
| Omarini 2019^31^ | pCR: Complete disappearance of invasive tumour in the breast and axillary lymph nodes.  Prognosis: OS - time from diagnosis of BC to death/last follow-up RFS - time from date of diagnosis to date of first documented relapse or death | Cut-off for high SFA and VFA was 100 (< 100 = normal; ≥ 100 = high adiposity).  Lumbar muscle cross-sectional area (LMCA, m2) used as measure of total body skeletal muscle mass (includes psoas, paraspinal and abdominal wall muscles)  Lumbar skeletal muscle index (LSMI, cm2/m2): total L3 skeletal muscle mass/body surface area. Sarcopenia cut-off = LSMI 38.5 (< 38.5 = sarcopenic; ≥ 38.5 = normal). Liver steatosis considered a measure of internal fat distribution. The CT liver-to-spleen ratio (L/S ratio) was used for fatty liver analysis.  Fatty liver defined as L/S ratio <1. |
| Rossi 2023^32^ | pCR: Radiological and clinical responses were assessed according to the Response Evaluation Criteria in Solid Tumours (RECIST). Patients were categorised into: 1. Responders: complete response (CR; no clinical evidence of residual tumour) and partial response (PR; reduction in size of the tumour more than 30%) 2. Non-Responders: stable disease (SD; reduction in size of the tumour inferior to 30%) and progressive disease (PD) | PecMA = cross-sectional area (square centimeters) of the average value for bilateral pectoralis muscles on MRI. |
| Tahtaci 2023^33^ |  | Cut-off value for sarcopenia = SMI 38.9 cm2/m2  Sarcopenic obesity = VFA divided by SMI |
| Trestini 2023^34^ | pCR: Absence of invasive tumour in the surgical specimen from the breast and axillary nodes (ypT0/is ypN0).  Prognosis: DFS - time from date of curative breast cancer surgery to date of breast cancer recurrence at local or regional sites, metastasis to distant sites, newly diagnosed breast cancer in the contralateral breast, other malignancies, or death from any cause. | SMI = SMM divided by body surface area (cm2/m2).  Cut-off value for sarcopenia in women: SMI <41 cm2/m2 |
| van den Berg 2019^37^ | Toxicity:  Toxicity-induced modifications to treatment - dose reductions, cycle delays, premature termination of chemotherapy, planned cytotoxic regime changed to another regime because of toxicities. Unknown reason for dose reduction or cycle delay (n=4) also included.  Logistical or other non-medical reasons for cycle delays were not classified as toxicity-induced modification of treatment | Pts categorised into tertiles of lean mass & fat mass:  Lowest tertile of lean mass - “low lean mass”  Upper two tertiles - “normal lean mass;”  Highest tertile of fat mass - “high fat mass”  Lower two tertiles as “normal fat mass.”  Based on this, created four groups:  (1) patients with a normal lean mass and normal fat mass,  (2) patients with a normal lean mass and high fat mass, (3) patients with low lean mass and normal fat mass, or (4) patients with low lean mass and high fat mass. |
| Yirgin 2020^36^ | pCR: Classified as  - The absence of invasive car­cinoma in breast tissue of the resected specimen.  - Residual ductal carcinoma in situ (DCIS) .  Pts grouped based on Miller-Payne grading system: Grade 1: no significant reduction in malignant cells;  Grade 2: minor loss of malignant cells (≤30%);  Grade 3: reduction in malignant cells between 30 - 90%;  Grade 4: disappearance of malignant cells >90%;  Grade 5: no malignant cells identifiable. | SAT - fat area superficial to the abdominal muscular wall  VAT - deep to muscular wall, consisting of the mesenteric, sub-peritoneal and retroperitoneal component MT - psoas, erector spinae, quadratus lumborum, transversus abdominis, and external oblique muscles. |

CI, confidence interval; CT, computed tomography; DFS, disease free survival; DDFS, distant disease-free survival; EFS, event-free survival; HU, Hounsfield unit; L/S ratio, liver/spleen ratio (surrogate for liver steatosis/internal fat distribution); NAC, neoadjuvant chemotherapy; NACI, neoadjuvant chemoimmunotherapy; OS, overall survival; pCR, pathological complete response; PFS, progression-free survival; PMA, psoas muscle area; RDI, relative dose intensity; RFS, recurrence-free survival; SAT, subcutaneous adipose tissue; SATI, subcutaneous adipose tissue index; SFA, subcutaneous fat area; SMA, skeletal muscle area; SMD, skeletal muscle density; SMI, skeletal muscle index; SUV, standardized uptake value; VAT, visceral adipose tissue; VFA, visceral fat area; VFI, visceral fat index; V/S ratio, VAT to SAT ratio.
